# Supplementary material for: Causal Relationship between Aging and Anorexia Nervosa: A White-Matter-Microstructure-Mediated Mendelian Randomization Analysis
Source: Biomedicines. 2024 Aug 16;12(8):1874. doi: 10.3390/biomedicines12081874 (PMC11351342; doi:10.3390/biomedicines12081874)
Supplement: Supplementary file 1 [file biomedicines-12-01874-s001.zip › Supplementary Figure/Figure S4/Singe SNP plot of MR analysis for TL to ubm-b-1653.pdf]

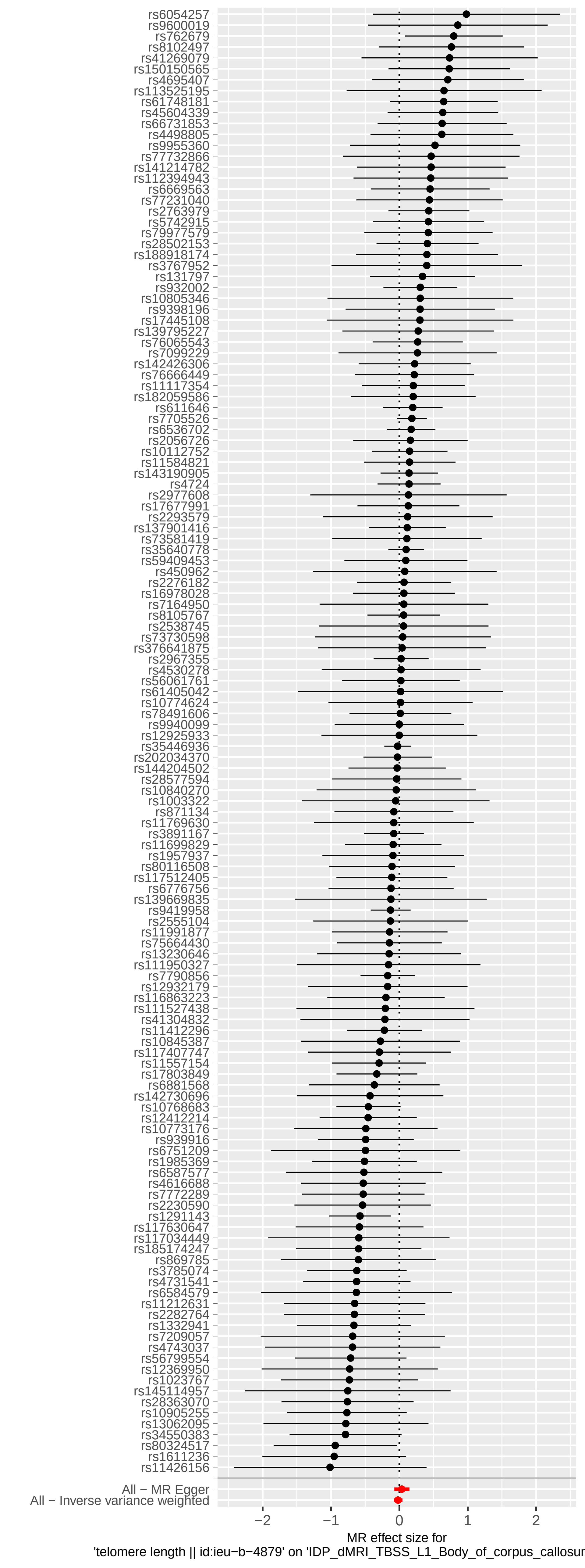
rs6054257
rs9600019
rs762679
rs8102497
rs41269079
rs150150565
rs4695407
rs113525195
rs61748181
rs45604339
rs66731853
rs4498805
rs9955360
rs77732866
rs141214782
rs112394943
rs6669563
rs77231040
rs2763979
rs5742915
rs79977579
rs28502153
rs188918174
rs3767952
rs131797
rs932002
rs10805346
rs9398196
rs17445108
rs139795227
rs76065543
rs7099229
rs142426306
rs76666449
rs11117354
rs182059586
rs611646
rs7705526
rs6536702
rs2056726
rs10112752
rs11584821
rs143190905
rs4724
rs2977608
rs17677991
rs2293579
rs137901416
rs73581419
rs35640778
rs59409453
rs450962
rs2276182
rs16978028
rs7164950
rs8105767
rs2538745
rs73730598
rs376641875
rs2967355
rs4530278
rs56061761
rs61405042
rs10774624
rs78491606
rs9940099
rs12925933
rs35446936
rs202034370
rs144204502
rs28577594
rs10840270
rs1003322
rs871134
rs11769630
rs3891167
rs11699829
rs1957937
rs80116508
rs117512405
rs6776756
rs139669835
rs9419958
rs2555104
rs11991877
rs75664430
rs13230646
rs111950327
rs7790856
rs12932179
rs116863223
rs111527438
rs41304832
rs11412296
rs10845387
rs117407747
rs11557154
rs17803849
rs6881568
rs142730696
rs10768683
rs12412214
rs10773176
rs939916
rs6751209
rs1985369
rs6587577
rs4616688
rs7772289
rs2230590
rs1291143
rs117630647
rs117034449
rs185174247
rs869785
rs3785074
rs4731541
rs6584579
rs11212631
rs2282764
rs1332941
rs7209057
rs4743037
rs56799554
rs12369950
rs1023767
rs145114957
rs28363070
rs10905255
rs13062095
rs34550383
rs80324517
rs1611236
rs11426156
All − MR Egger
All − Inverse variance weighted
−2
−1
0
1
2
MR effect size for
'telomere length || id:ieu−b−4879' on 'IDP_dMRI_TBSS_L1_Body_of_corpus_callosur
